# Long-term Dalbavancin for Suppression of Gram-Positive Chronic Left Ventricular Assist Device Infections

**DOI:** 10.1093/ofid/ofad537

**Published:** 2023-11-02

**Authors:** Sarah Rowe, Sarah Green, Benjamin Albrecht, Stephanie M Pouch

**Affiliations:** Emory University Hospital Pharmacy Department, Atlanta, Georgia, USA; Emory University Hospital Pharmacy Department, Atlanta, Georgia, USA; Emory University Hospital Pharmacy Department, Atlanta, Georgia, USA; Emory University Hospital Pharmacy Department, Atlanta, Georgia, USA; Emory University School of Medicine Department of Medicine, Atlanta, Georgia, USA

**Keywords:** MRSA, dalbavancin, gram-positive, left ventricular assist device, suppression

## Abstract

**Background:**

Infection is the leading cause of morbidity and mortality in patients with left ventricular assist devices (LVADs). Prolonged suppressive therapy should be strongly considered and is often used in patients with recurrent infections when source control cannot be achieved. Dalbavancin is a promising option in patients with LVADs requiring prolonged durations of antibiotic therapy, especially when no oral alternatives are available.

**Methods:**

This case series included 8 patients receiving dalbavancin for the long-term suppression of gram-positive infections at Emory University Hospital and Emory St Joseph's Hospital.

**Results:**

The overall incidence of breakthrough infections occurred in 5 of the 8 patients included in the study. One patient experienced an early breakthrough infection within 1 month of dalbavancin initiation. Another experienced a breakthrough infection within 3 and 6 months of dalbavancin initiation, and the final 3 patients experienced a breakthrough infection within 6 and 12 months. The average duration of dalbavancin suppression therapy among all patients was 229 days, and no adverse effects were reported.

**Conclusions:**

Dalbavancin is a promising option in patients who require long-term suppression for chronic gram-positive LVAD infections, given its unique pharmacokinetic profile and excellent tissue penetration. The use of biweekly dalbavancin infusions in our 8 patients prevented infection for an extended period of time despite some of the patients not being able to consistently receive infusions. Larger studies are needed to determine the efficacy and safety of using dalbavancin for long-term suppression of gram-positive LVAD infections.

## Teaser

Dalbavancin is a promising option for the suppression of chronic gram-positive left ventricular assist device infections. The use of dalbavancin in our 8 patients provided prolonged suppression without the incidence of adverse effects.

A left ventricular assist devices (LVAD) is a form of life support that is often used in patients with end-stage heart failure as either a bridge to heart transplantation or as destination therapy. Studies have shown that infection is among the most common adverse effects and has been reported in up to 37% of patients within the first year of device placement [1–3]. The majority of microorganisms responsible for these infections include gram-positive skin flora, such as *Staphylococcus aureus,* coagulase-negative *Staphylococcus*, and pyogenic *Streptococcus* [3]. Prolonged suppressive therapy is often used in patients with recurrent infections when source control cannot be achieved [2].

Dalbavancin, a long-acting lipoglycopeptide antibiotic that inhibits cell wall synthesis, is a promising option in patients with LVADs requiring prolonged durations of parenteral antibiotic therapy. Dalbavancin has a terminal half-life of 14.4 days and is currently approved by the Food and Drug Administration for the treatment of acute bacterial skin and skin structure infections [4]. Dalbavancin has also exhibited potent activity against gram-positive organisms in bone and joint infections [5]. In addition, its long half-life allows for weekly or biweekly infusions, making it an ideal option for outpatient antibiotic therapy [6].

Emory Healthcare currently uses dalbavancin as an option for suppression in patients with chronic drive-line infections. As many of these patients are not candidates for transplantation, several months of suppression with dalbavancin may be used. The practice of using dalbavancin for infection suppression in patients with LVADs as multiple, repeated doses of dalbavancin over several months is unique and has shown to provide prolonged suppression in numerous patients [7, 8]. We present a case series of 8 patients with chronic gram-positive LVAD-related infections receiving dalbavancin for suppression.

## METHODS

This case series was conducted via retrospective record review of inpatient and outpatient infectious disease consultations and infusion appointments for patients with LVADs who received dalbavancin between 1 July 2017 and 30 November 2022. Patients were included if they were >18 years of age, received dalbavancin for suppression of chronic LVAD infections, and were retained in the Emory Healthcare system. Patients were excluded if they received dalbavancin for treatment of infection without subsequent continuation for suppression or received <2 consecutive doses of dalbavancin after LVAD implantation.

Data related to patient demographic information, renal function, LVAD indication, previous LVAD infection suppression regimens, and dalbavancin administrations were obtained. Dalbavancin-related adverse effects, and the reason for dalbavancin discontinuation were also recorded, as applicable. Dalbavancin-related adverse effects were assessed from the transplant infectious diseases clinic notes. Patient records were also reviewed for follow-up visits with the transplant infectious diseases clinic, infusion appointments, and readmissions to Emory University Hospital and Emory St Joseph's Hospital. This research did not include factors necessitating patient consent.

The overall incidence of breakthrough infections was recorded, as well as breakthrough infections at 1, 3, 6, and 12 months. A breakthrough infection was defined as the incidence of positive tissue, wound, or blood culture after the initiation of dalbavancin.

## RESULTS

Of a total of 127 patients screened, 119 were excluded because they were receiving dalbavancin for the treatment of either a non–LVAD-related or LVAD-related infection. Five of the 8 patients meeting inclusion criteria experienced a gram-positive breakthrough infection. One patient experienced an early breakthrough infection within 1 month after dalbavancin initiation, another patient experienced a breakthrough infection between 3 and 6 months after dalbavancin initiation, and the final 3 patients experienced a breakthrough infection between 6 and 12 months. On record review, there were no reported dalbavancin-related adverse effects. The average duration of suppression therapy was 229 days in our 8 patients, with durations ranging from 107 to 372 days. Transition to oral suppression, treatment of breakthrough infections, LVAD replacement, heart transplantation, and national dalbavancin shortage were among the reasons that dalbavancin suppression was discontinued. Baseline characteristics, including previous suppression regimens, organisms isolated before dalbavancin initiation, and type of LVAD at dalbavancin initiation, are included in Table 1, along with a summary of each patient's clinical course.

**Table 1.** Dalbavancin Summary Table

Characteristic	Patient							
	1	2	3	4	5	6	7	8
Age at DAL initiation, y/sex	51/M	31/F	40/M	37/F	57/M	64/F	52/M	57/M
Weight, kg	156	128	119	62	76	86	94	103
CrCl at DAL initiation, mL/min	57.1	58.9	18	90	63	32	71	116
Type of LVAD at time of DAL initiation	Heartmate III	Heartmate III	Heartmate III	Heartware	Heartmate III	Heartmate II	Heartmate II	Heartmate III
Reason for LVAD implantation	NICM	PPCM	NICM	PPCM	NICM	ICM	ICM	NICM
LVAD for BTT or DT	DT	DT	BTT	DT	DT	DT	DT	DT
Gram-positive organism(s) identified with previous infection	MRSA	*Staphylococcus lugdunensis* and *Staphylococcus epidermidis*	MRSA	MRSA	MRSA and *C. Striatium*	MSSA	MRSA	*S epidermidis* and *Corynebacterium jeikeium*
Other organism(s) identified with previous infection	None	None	*Pseudomonas* spp	*Proteus mirabilis*	None	None	None	None
Previous suppression regimen(s)	Doxycycline	Doxycycline and SMX/TMP	Vancomycin	Doxycycline	Doxycycline and minocycline	Cephalexin, doxycycline, SMX/TMP, and linezolid	SMX/TMP and doxycycline	None
Reason for switching suppression to DAL	Breakthrough infection	Breakthrough infection	Breakthrough infection	Breakthrough infection	Breakthrough infection	Adverse effects to oral suppression	Breakthrough infection	NA
Frequency of DAL administration	Biweekly	Biweekly	Biweekly	Biweekly	Biweekly	Weekly, switched to biweekly after 2 mo	Biweekly	Biweekly
Reason for switching DAL to another agent, if applicable	Breakthrough infection	Breakthrough infection	NA	Breakthrough infection	Breakthrough infection	Clinically stable	Breakthrough infection	Clinically stable
Agent DAL was switched to	Linezolid, ceftaroline	Linezolid	NA	Daptomycin and ceftaroline	Vancomycin and daptomycin	Doxycycline	Daptomycin	Minocycline
Gram-positive organism(s) identified with breakthrough infection, if applicable	MRSA	MRSA	NA	MRSA	*C. Striatium*	NA	MRSA	NA
Time to first LVAD-related infection after DAL suppression initiation, d	112	273	NA	226	251	NA	29	NA
Total duration of DAL for suppression, d	127	273	212	226	372	227	293	107
Missed or delayed infusions	No	No	No	Yes	Yes	No	No	No
Outcome	Continued	Continued	Heart transplantation	LVAD exchange and oral suppression	Continued	Oral suppression	LVAD exchange and oral suppression	Oral suppression

Abbreviations: BTT, bridge to transplant; CrCl, creatinine clearance; DAL, dalbavancin; DT, destination therapy; F, female; ICM, ischemic cardiomyopathy; LVAD, left ventricular assist device; M, male; MRSA, methicillin-resistant *Staphylococcus aureus*; MSSA, methicillin-susceptible *S aureus*; NA, not applicable; NICM, nonischemic cardiomyopathy; PPCM, peripartum cardiomyopathy; SMX/TMP, sulfamethoxazole-trimethoprim.

## CASE REPORTS

### Patient 1

A 51-year-old man with a medical history of nonischemic cardiomyopathy (NICM) underwent a Heartmate III LVAD implantation as destination therapy. This patient presented to Emory University Hospital with purulent drive-line exit site (DLES) drainage with wound cultures positive for methicillin-resistant *S aureus* (MRSA). He was initially treated with vancomycin; however, an acute kidney injury developed, with consistently supratherapeutic vancomycin troughs, and the patient was transitioned to daptomycin for the remainder of his treatment course. After completion of daptomycin therapy, the patient was discharged on doxycycline for long-term suppression.

Unfortunately, the patient experienced numerous breakthrough infections while on doxycycline. He later presented to our institution with his third MRSA breakthrough infection while consistently taking doxycycline, and he was treated with vancomycin, ceftaroline, and tedizolid during his inpatient stay. At discharge, doxycycline was discontinued, and treatment with intravenous dalbavancin 1500 mg every 2 weeks was initiated for long-term suppressive therapy. The patient presented to the transplant infectious diseases clinic for follow-up with purulent drainage from his DLES, despite consistently receiving his scheduled dalbavancin infusions. The wound cultures obtained from the DLES were positive for MRSA. He was admitted to Emory University Hospital and treated with linezolid and ceftaroline for a total of 6 weeks. On completion of breakthrough infection treatment, suppressive biweekly dalbavancin infusions were resumed, which continue at this writing.

### Patient 2

A 30-year-old woman with a medical history of NICM secondary to peripartum cardiomyopathy underwent a Heartware LVAD implantation for destination therapy. She was admitted with purulent drainage from the DLES. Wound cultures obtained from the DLES were positive for *Staphylococcus lugdunensis,* for which the patient was treated with cefadroxil. She later presented with *Staphylococcus epidermidis* bacteremia, with the source presumed to be from the DLES. She was treated with vancomycin for a total of 28 days. On completion of the vancomycin, doxycycline was initiated for long-term suppression. However, the patient experienced significant gastrointestinal upset on this suppression regimen and was transitioned to sulfamethoxazole-trimethoprim (SMX/TMP).

The patient later had her Heartware LVAD explanted and a Heartmate III LVAD implanted owing to pump thrombosis. She experienced 2 breakthrough infections while on SMX/TMP for suppression after the Heartmate III LVAD was placed. After the second breakthrough infection, she was treated with daptomycin for 4 weeks, followed by dalbavancin 1500-mg biweekly infusions for suppression. The patient later presented to the transplant infectious diseases clinic for a follow-up visit with increased drainage from her DLES and was found to have a MRSA breakthrough infection. She was treated with 2 weeks of oral linezolid, followed by resumption of biweekly dalbavancin infusions. She remains on dalbavancin suppression at this writing.

### Patient 3

A 40-year-old man with a medical history of NICM underwent a Heartmate III LVAD implantation as a bridge to heart transplantation. He later presented to our institution with acute decompensated heart failure and was listed for expedited heart transplantation. At that time, a history of a MRSA drive line and *Pseudomonas* spp pocket infection treated at an outside hospital was noted. During his admission at Emory University Hospital, the patient was found to have persistent *Pseudomonas* spp bacteremia, for which he was treated with ceftazidime. The patient was discharged on indefinite ceftazidime and dalbavancin 1500-mg biweekly infusions for long-term *Pseudomonas* and MRSA suppression, respectively. The patient later underwent open-heart transplantation at an outside hospital and both *Pseudomonas* and MRSA suppression therapy was discontinued.

### Patient 4

A 36-year-old woman with a medical history of NICM secondary to peripartum cardiomyopathy underwent a Heartware LVAD implantation as destination therapy. This patient presented to our transplant infectious diseases clinic with significant drainage from her DLES, and MRSA was isolated. She was subsequently admitted to Emory University Hospital for treatment and received vancomycin for a total of 6 weeks, followed by doxycycline for long-term suppression. She was admitted to our institution again with another MRSA bacteremia and drive-line infection, and she was treated with daptomycin followed by dalbavancin 1500-mg biweekly infusions for long-term suppression.

The patient remained on this regimen until she presented to our institution with her first MRSA breakthrough infection in the setting of missed dalbavancin infusions. She was treated with daptomycin for a total of 6 weeks, followed by resumption of biweekly dalbavancin infusions for suppression. Of note, the patient had received the majority of her infusions at an outside hospital, and our team was unable to assess the exact dates of those infusions.

The patient later presented again to our institution with her second episode of MRSA bacteremia, for which she was treated with daptomycin, followed by resumption of dalbavancin for suppression. A month later, she presented to our institution with her third MRSA and *Proteus mirabilis* breakthrough infection. During this stay, she was found to have 2 different strains of MRSA, both of which were resistant to daptomycin. She was treated with ceftaroline for 6 weeks and ultimately had her Heartware LVAD explanted and a Heartmate III LVAD implanted. Dalbavancin biweekly infusions for suppression were discontinued, and doxycycline and cefpodoxime were initiated for long-term suppression.

### Patient 5

A 57-year-old man with a medical history of NICM underwent a Heartmate III LVAD implantation as destination therapy. This patient was later admitted to Emory University Hospital with purulent drainage from his DLES, in which MRSA was isolated, and he was treated with a 14-day course of doxycycline. One month later, he was admitted to our institution and found to have a *Corynebacterium striatum* DLI and bacteremia, for which he was treated with vancomycin, followed by doxycycline for long-term suppression.

The patient was later admitted again to our institution with *C striatum* bacteremia, and computed tomography revealed multiple peri­–drive-line abscesses. Vancomycin was originally initiated for a planned total duration of 6 weeks, but after an acute kidney injury developed the patient was switched to daptomycin for the remainder of the treatment course. On completion of treatment, 1500-mg biweekly infusions of dalbavancin were initiated for long-term suppression. Of note, the patient had many extended gaps (>3 weeks) between dalbavancin infusions.

A few months later, the patient presented to Emory University Hospital with worsening drive-line infection and multiple abscesses, despite treatment with vancomycin and ceftazidime. He underwent incision and drainage of the abscesses, and his treatment regimen was changed to meropenem and daptomycin. No organisms were isolated from the fluid cultures obtained during the procedure. However, 16S rRNA test results were positive for *C striatum.* The patient was discharged on daptomycin and meropenem.

This patient was admitted again 1 month later with bloody, purulent drainage from his drive-line site and was found to have another *C striatum* and *Pseudomonas* infection. He was treated with vancomycin and ceftazidime, followed by resumption of dalbavancin and levofloxacin for suppression, which continues at this time.

### Patient 6

A 59-year-old woman with a medical history of ischemic cardiomyopathy underwent a Heartmate II LVAD implantation as destination therapy. She patient experienced no issues with her LVAD until she presented to Emory University Hospital with a methicillin-susceptible *S aureus* DLES infection 2 years later. She was treated with cephalexin and instructed to continue taking it for suppression at that time. According to outside records, it appeared that this patient transferred her care after this admission and was transitioned to doxycycline for suppression. She was admitted again to our institution and found to have an MRSA drive-line infection that was resistant to tetracyclines, requiring transition to vancomycin, followed by SMX/TMP for suppression. Shortly after the initiation of SMX/TMP, the patient experienced fevers, acute kidney injury, and hepatitis, and she was switched to linezolid. Owing to the potential adverse effects associated with long-term linezolid use, the transplant infectious diseases clinic transitioned the patient to dalbavancin 1500-mg weekly infusions for suppression.

The patient received weekly infusions of dalbavancin for approximately 2 months then was switched to a biweekly regimen, on which she remained for an additional 4 months. She remained clinically stable and no longer experienced drive-line site issues after approximately 6 months of dalbavancin therapy, so she was transitioned back to oral doxycycline for long-term suppression, which continues at this time.

### Patient 7

A 48-year-old man with a medical history of dilated cardiomyopathy underwent a Heartmate II LVAD implantation as destination therapy. The patient first presented to Emory University Hospital, where he reported taking SMX/TMP for long-term MRSA suppression. He later experienced pump thrombosis and underwent a pump exchange, at which time SMX/TMP suppression therapy was discontinued. From an infectious standpoint, the patient’s condition remained stable for a few years until he presented to our institution with fevers, upper abdominal pain, and purulent DLES drainage. Blood cultures were obtained, and computed tomography revealed multiple soft-tissue abscesses along the drive line tract and sternum. MRSA was isolated from the blood cultures, and the patient was treated with daptomycin for 6 weeks.

The patient later presented to our institution again with increased drainage from his DLES. Blood cultures obtained during his stay were positive for MRSA. He underwent incision and drainage of the abscesses and was discharged on daptomycin and ceftaroline treatment, followed by doxycycline for suppression. A few months later, he presented with another MRSA bacteremia, for which he was treated with ceftaroline, followed by 1500-mg biweekly infusions of dalbavancin for long-term suppression.

The patient remained suppressed for approximately 4 months until he presented to our institution with his first MRSA breakthrough infection despite consistently receiving dalbavancin infusions. After receiving treatment for the breakthrough infection, he was instructed to resume dalbavancin as well as oral doxycycline, 100 mg twice daily for suppression.

The patient remained suppressed on dual gram-positive suppression for approximately 3 months until he presented with his second MRSA breakthrough infection, for which he was treated with vancomycin, followed by resumption of dalbavancin. Ultimately, the patient had his Heartmate II LVAD explanted and was temporarily maintained on a continuous milrinone pump. Despite having his LVAD explanted and consistently receiving dalbavancin, he continued to have purulent drainage from his sternal wound and was found to have his third MRSA breakthrough infection. The patient was treated with daptomycin, underwent a Heartmate III LVAD implantation, and was started on doxycycline for long-term suppression, which continues at this time.

### Patient 8

A 57-year-old man with a medical history of NICM underwent a Heartmate III LVAD implantation as destination therapy. He presented to our institution 1 month later with sternal wound drainage and cultures positive for *S epidermidis,* for which he was treated with a 7-day course of cephalexin. The patient later presented to our institution with chest pain, shortness of breath, and purulent drainage from his DLES. *Corynebacterium jeikeium* was isolated from his wound cultures, and he was treated with doxycycline for 7 days. This patient was admitted again a few months later with a second *C jeikeium* infection and was treated with vancomycin. On discharge, dalbavancin 1500-mg biweekly infusions were initiated. The patient remained on this regimen for approximately 107 days until he transitioned to oral minocycline, as he remained clinically stable and preferred an oral suppression regimen. He did not experience a gram-positive breakthrough infection at any time point while receiving dalbavancin and remains on oral suppression at this time.

## DISCUSSION

We present 8 patients with chronic gram-positive LVAD-related infections who received 1500-mg dalbavancin infusions for suppressive therapy. All 8 patients had received previous suppressive therapy with intravenous or oral antibiotics before dalbavancin initiation. Patients 3, 6, and 8 did not experience breakthrough infection at any time while receiving dalbavancin. Patients 4 and 5 experienced breakthrough infections in the setting of missed dalbavancin infusion appointments. However, patients 4 and 5 remained suppressed for a total of 226 and 251 days, respectively, before experiencing a breakthrough infection. Finally, patients 1, 2, and 7 experienced numerous breakthrough infections despite consistently receiving dalbavancin infusions.

Dalbavancin provided suppression for an average of 229 days in our patients, which is comparable to the average of 175 days which has been reported with oral suppression therapy [9, 10]. The exact dosing of dalbavancin for this indication has not yet been established. Our 8 patients received 1500 mg of dalbavancin on a weekly, biweekly, or monthly basis. The majority of our patients received dalbavancin on a biweekly basis, but in 1 patient monthly dosing of dalbavancin provided suppression for an extensive period of time as well.

Current literature available is limited regarding long-term antimicrobial suppression recommendations in this patient population. Overall, dalbavancin may be a promising option in patients who require long-term suppression for chronic gram-positive LVAD-related infections given its extensive half-life and minimal adverse drug effects. The use of dalbavancin should be considered in this patient population, especially when other intravenous and oral suppression regimens have failed. Our study contributes to the available data supporting the use of dalbavancin for this indication, but larger studies are needed to determine the efficacy and safety of using dalbavancin for long-term suppression of gram-positive LVAD infections.
